# The Application of Indium Oxide@CPM-5-C-600 Composite Material Derived from MOF in Cathode Material of Lithium Sulfur Batteries

**DOI:** 10.3390/nano10010177

**Published:** 2020-01-20

**Authors:** Guodong Han, Xin Wang, Jia Yao, Mi Zhang, Juan Wang

**Affiliations:** Shanxi Key Laboratory of Nanomaterials & Nanotechnology, School of Mechanical & Electrical Engineering, Xi’an University of Architecture & Technology, Xi’an 710055, China; a18829342832@126.com (G.H.); xinwang1107@163.com (X.W.); flurenceyao@outlook.com (J.Y.); chriszhang7856@outlook.com (M.Z.)

**Keywords:** shuttle effect, CPM-5, indium oxide, core-shell, coulombic efficiency

## Abstract

Due to the “shuttle effect”, the cycle performance of lithium sulfur (Li-S) battery is poor and the capacity decays rapidly. Replacing lithium-ion battery is the maximum problem to be overcome. In order to solve this problem, we use a cage like microporous MOF(CPM-5) as a carbon source, which is carbonized at high temperature to get a micro-mesoporous carbon composite material. In addition, indium oxide particles formed during carbonization are deposited on CPM-5 structure, forming a simple core-shell structure CPM-5-C-600. When it is used as the cathode of Li-S battery, the small molecule sulfide can be confined in the micropores, while the existence of large pore size mesopores can provide a channel for the transmission of lithium ions, so as to improve the conductivity of the material and the rate performance of the battery. After 100 cycles, the specific capacity of the battery can be still maintained at 650 mA h·g^−1^ and the Coulombic efficiency is close to 100%. When the rate goes up to 2 C, the first discharge capacity not only can reach 1400 mA h·g^−1^, but also still provides 500 mA h·g^−1^ after 200 cycles, showing excellent rate performance.

## 1. Introduction

With the increasing demand for electric vehicles and high scale-energy storage, searching for the high-performance energy storage materials has become a new focus [1,2]. Lithium-ion (Li^+^) batteries have become an indispensable part of various electronic products because of high voltage and good stability [3,4,5,6,7,8,9]. However, the Li^+^ battery has such problems as low capacity and poor safety, which greatly limits its application. Therefore, the next generation battery, Lithium-sulfur (Li-S) battery, which incorporate abundant active sulfur as cathode materials, has drawn extensive research as one of the most promising candidates for next generation energy storage system. Owing to the high theoretical specific capacity of 1675 mA h·g^−1^ (fourfold higher than the conventional state-of-the-art LIBs) and energy density of 2600 Wh kg^−1^ [10], it can replace Li^+^ battery. However, although sulfur has many advantages, such as low cost, abundant natural resources and low toxicity, several issues need to be solved for sulfur as a cathode for practical application of Li-S battery. First, during the process of charging and discharging, Li-S batteries have a large volume expansion, and it is easy to cause damage to the structure of the negative electrode material during the cycle; Second, the poor electronic conductivity of element S (5 × 10^−30^ cm^−1^ at 25 °C) can affect the conductivity of materials and the rate performance of batteries; Third, During the cycle, the production of Li_2_S and Li_2_S_2_ will coat the surface of the active material, thereby preventing Li^+^ from entering the electrode; Fourth, the intermediate products (polysulfides) of the reaction easily dissolve in the electrolyte, which will cause severe capacity decay, resulting in a serious “shuttle effect” [11,12,13,14].

In order to solve these problems, a combination of carbon materials and sulfur is used to form a positive electrode to effectively improve the electrochemical performance of Li-S batteries. The carbon materials such as, porous carbon, carbon nanospheres, microporous and mesoporous carbon [15,16,17,18]. The combination of these carbon materials and sulfur can solve the problem of poor conductivity of elemental sulfur and improve the rate performance of Li-S batteries. However, due to the defects of the pore structure and the material itself, the cycle stability of the composite material as the positive material of Li-S batteries is not ideal [19]. So finding the positive carrier material with good conductivity and cycle stability has become a focus problem for many researchers. In recent years, due to their large specific surface area, adjustable pores and good electrical conductivity, porous carbon materials prepared by directly carbonization of metal-organic framework (MOF) have been widely used in the fields of drug delivery and energy storage equipment [20]. So, selecting suitable MOFs with high thermal stability and making them become the carbon sources will prove to be an effectively method to overcome the shortcomings of the Li-S battery. Novel porous carbon materials with unique structures are promising candidates for immobilizing sulfur as cathode for Li-S batteries.

In this work, we choose a typical In-based MOF (CPM-5) as an initial precursor [21]. CPM-5 has a unique and attractive three-dimensional structure, which makes it have high carbon dioxide (CO_2_) adsorption capacity, thermal and water stability. This is because there are two valence states in its structure, namely, one in the form of a trinuclear cluster with a positive charge, the other in the form of a single metal with a negative charge. The combination of them turns CPM-5 into a special cage structure. Therefore, the In-MOF (CPM-5) is carbonized directly in the furnace at 600 °C under N_2_ gas protection, resulting in a large specific surface area and micro-mesoporous structure. The indium oxide particles formed in the carbonization process are combined with CPM-5 to form a core-shell structure. The existence of this structure will reduce the loss of active substances and improve the conversion efficiency of materials. When used as a positive electrode for Li-S batteries, they have high reversible capacity, high coulombic efficiency, and excellent rate performance [22,23].

## 2. Experimental Section

### 2.1. Materials Preparation

In(NO_3_)_3_·xH_2_O, Trimesic acid (H_3_BTC), N,N-Dimethylformamide(DMF), Deionized water(H_2_O), Sulfur (S), Acetylene black (AB), N-Methyl-2-pyrrolidone (NMP), Polyvinylidene fluoride (PVDF) and 1 M LiTFSI in Ethylene glycol dimethyl ether (DME) and 1,3-Dioxolane (DOL) (v:v = 1:1) with 1 wt% LiNO_3_. All chemicals and solvents employed were obtained from commercial sources and used as received without further purification.

### 2.2. Synthesis of CPM-5 Materials

A mixture of In(NO_3_)_3_·xH_2_O (0.20 g), and H_3_BTC (0.17 g) was stirred in a mixed solution of H_2_O/DMF (1.0 g/4.0 g) for 1 h, and then transferred into a Teflon-lined stainless steel autoclave (23 mL). The Teflon-lined stainless steel autoclave was kept at 120 °C for 5 days, and then cooled to room-temperature. After washed by water and ethanol, the colorless crystals were obtained. CPM-5 crystals are activated by drying in a vacuum oven at 100 °C for 12 h.

### 2.3. Synthesis of CPM-5-C-600 Materials

The dried CPM-5 powders were put into a porcelain boat and heated at 600 °C for 4 h, with a heating rate of 5.0 °C∙min^−1^ in a N_2_ filled tubular furnace. Subsequently, the samples were cooled down to room temperature in the furnace. The black precipitates were then collected.

### 2.4. Synthesis of CPM-5-C-600@S Materials

Using the masses of CPM-5-C-600 and Pure S,S: CPM-5-C-600 loading ratios of 1:1 was used. The sulfur and CPM-5-C-600 powders were grinded with the mortar for 30 min in the glovebox filled with argon. Then the mixture was transferred to the tube furnace and heated at 155 °C for 12 h to allow the melt sulfur immersed into the CPM-5-C-600 pores. After cooling to room temperature, the CPM-5-C-600@S compound materials were obtained. The synthetic method of cathode materials AB@S, CPM-5@S adopted exactly the same experimental method.

### 2.5. Materials Characterization

All the samples were characterized using Power X-ray diffraction (Japan Rigaku D/Max2550VB+/PC diffractometer equipped with Cu Kα radiation (λ = 1.5406 Å) at a scan rate of 2° in the range of 5°–70°. Scanning electron microscopy (SEM, JEOL 6700F, 5 keV) equipped with an energy dispersive X-ray analyzer (EDS, EDX Genesis 4000 X-ray Analysis System), Thermogravimetric analysis (TGA) was conducted using a NETSCHZ STA-449C thermal analyzer under flowing N_2_ at a heating rate of 5.0 °C min^−1^. N_2_ sorption measurements were performed using Micromeritics 3-Flex surface-area and a pore-size analyzer instrument at 77 K. All samples were activated at 80 °C in vacuum for 12 h before tests. The surface area of the materials was calculated based on the Brunauer-Emmett-Teller (BET) method and the pore size distribution was calculated based on the Barrett-Joyner-Halenda (BJH) model.

### 2.6. Cells Assembly and Electrochemical Measurements

The cathode slurry was prepared using a 50% wt CPM-5-C-600@S, 30% wt AB and 20% wt PVDF solid mixture in NMP. Firstly, CPM-5-C-600@S and AB were grinded uniformly for 30 min, then poured into NMP solution which had dissolved PVDF, and stirred by magnetic force for 12 h. Then, the slurry was coated onto aluminum foil substrate. After vacuum drying at 60 °C for 12 h, the electrode was obtained and transferred to an argon-filled glovebox (MBRAUN LABSTAR, H_2_O ≤ 0.1 ppm, O_2_ ≤ 0.1 ppm) with Li as the counter electrode and polypropylene film as the separator for cell assembly. The electrolyte was composed of 1 M LITFSI in a mixed solution of DME and DOL (1:1) with an added 1 wt% LiNO_3._

Cyclic Voltammetry (CV) and Electrochemical Impedance Spectroscopy (EIS) tests were carried out using a CHI660E electrochemical workstation (Shanghai Chenhua Instrument, Shanghai, China). The CV measurement was performed between 1.6 and 2.9 V vs. Li^+^/Li at a scanning rate of 0.1 mV∙s^−1^. The EIS was conducted at open-circuit condition with a frequency range from 10^−2^ to 10^5^ Hz with the amplitude of 5 mV. The charge-discharge cycling test were worked within voltage window of 1.6 to 2.9 V vs. Li^+^/Li using a battery testing system NEWARE-BTS-CT4008-5 V 10 mA (Shenzhen neware electronics, Shenzhen, China) at various rates.

## 3. Results and Discussions

### 3.1. Characteristics

**Figure 1:**
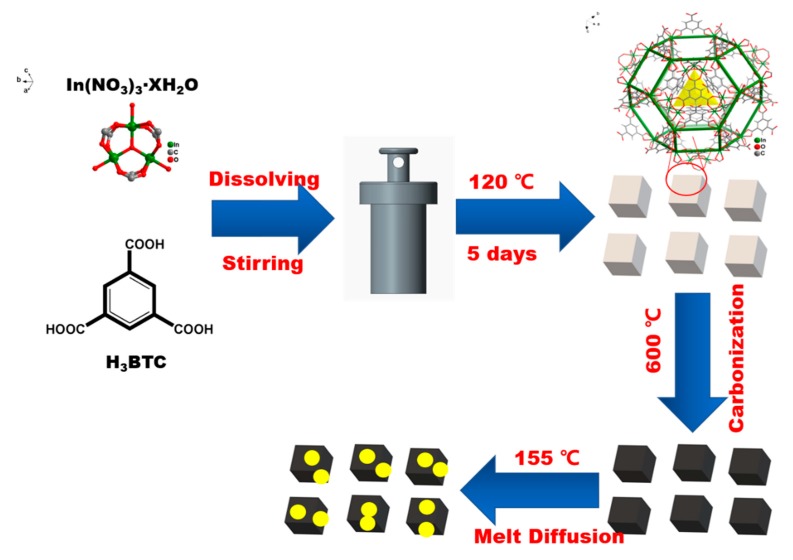
The Schematic illustration of the CPM-5-C-600@S composite preparation.

**Figure 2:**
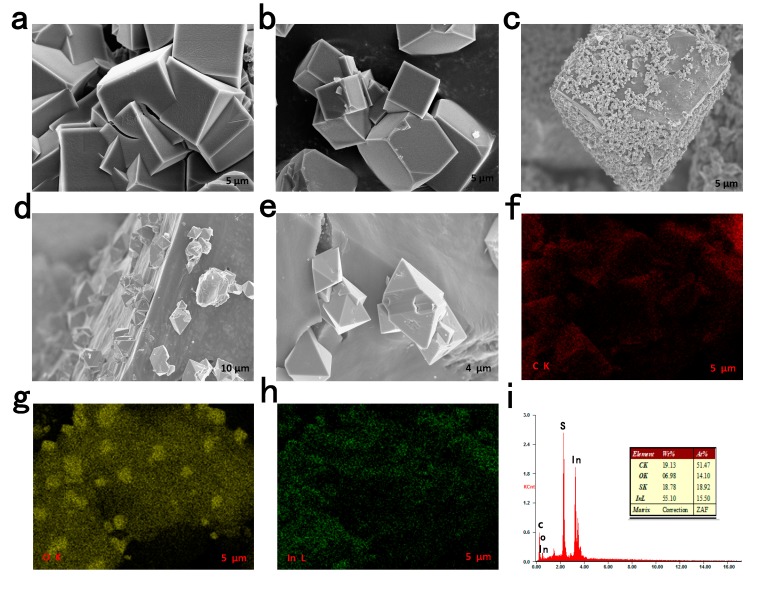
SEM images of: (**a**,**b**) CPM-5 and (**c**–**e**) CPM-5-C-600. (**f**–**h**) The elemental mapping of Indium oxide particles of CPM-5-C-600. (**i**) The corresponding EDX of CPM-5-C-600@S.

The CPM-5-C-600 was prepared through one-step carbonization of CPM-5, as illustrated in Figure 1. Firstly, CPM-5 crystal particles were synthesized by hydrothermal method at 120 °C in the reactor. The obtained crystal was not treated except for simple drying. Then, CPM-5-C-600 was achieved by high temperature carbonization at 600 °C. The SEM (Scanning electron microscopy) of CPM-5, CPM-5-C-600 and the elemental mapping of CPM-5-C-600@S are shown in Figure 2. Figure 2a,b is the morphology of CPM-5. It can be seen that CPM-5 has a regular cube structure, which is consistent with what had been reported previously. After high temperature carbonization at 600 °C, the morphology of CPM-5-C-600 is shown in Figure 2c–e. CPM-5-C-600 maintained the original cube shape, which also demonstrated the thermal stability of CPM-5 structure. However, there are some small rhombic crystals, which are attached to the surface of CPM-5-C-600. It is assumed that a part of Indium on the surface of CPM-5 structure is transformed into indium oxide at high temperature and deposited on the undamaged CPM-5. In addition to the presence of carbon, we found the indium and oxygen elements were distributed on the surface of CPM-5-C-600 uniformly after the EDS analysis and test. We didn’t choose to wash off the metal oxides on the surface with strong acid or alkali, because we supposed that the existence of metal oxides would form a specific core-shell structure with CPM-5-C-600 [24]. Therefore, the composite material will not only enhance the conductivity of the material and improve the rate performance of the battery, but also inhibit the intermediate product polysulfide, which will decrease the decay rate of polysulfide and promote the cycle stability of the battery [25]. To further characterize the sulfur distribution in the CPM-5-C-600@S, the EDX of CPM-5-C-600@S at the selected area is shown in Figure 2i, proving that the sulfur was homogenously absorbed within the CPM-5-C-600 host [26].

**Figure 3:**
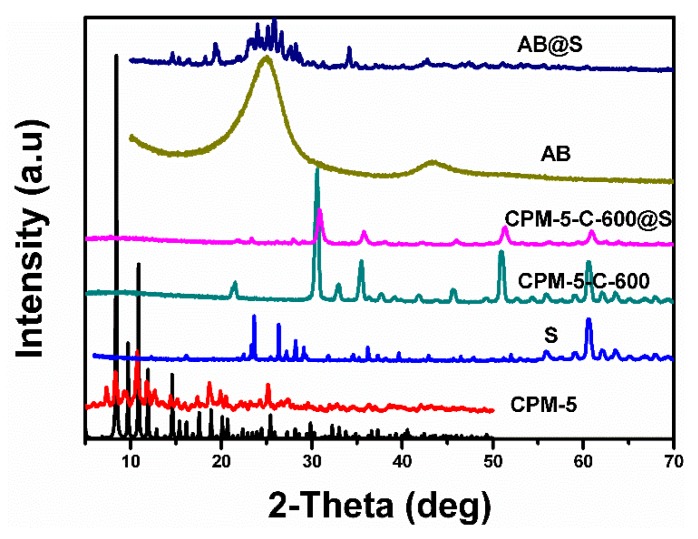
The XRD patterns ofCPM-5, S, AB, AB@S, CPM-5-C-600 and CPM-5-C-600@S composites.

Powder X-ray diffraction patterns of the CPM-5, CPM-5-C-600, CPM-5-C-600@S, AB, and AB@S are shown in Figure 3. By comparing with the standard card, a large quantity of pure CPM-5 (gram-scale) can be readily prepared. The characteristic peak of CPM-5 can be seen between 5° and 10° obviously, which shows its good crystallinity. There is no impurity peak that indicates its good purity [21]. For AB, sharp diffraction peaks appearing near 26° and 44° are the characteristics of the carbon materials [26]. The peaks corresponding to the sulfur were observed from AB@S composite, indicating that the sulfur particles are distributed to the materials uniformly [27]. On the contrary, there were no obvious peaks appearing in the XRD profile of CPM-5-C-600, suggesting comparatively low graphite degree of the porous carbon derived from CPM-5 because of a low carbonization temperature [27,28]. Besides, the peaks at 31°, 36° and 52° have very strong diffraction intensity, which are the feature peaks of indium oxide [29]. This also verified our conjecture about indium oxide in SEM analysis. This indicates that central indium exists in MOF in the form of metal oxides after carbonization. The peaks of sulfur were not found in CPM-5-C@S, implying that the confining of sulfur within the CPM-5-C-600 pores is due to a relatively large pore volume and specific area.

**Figure 4:**
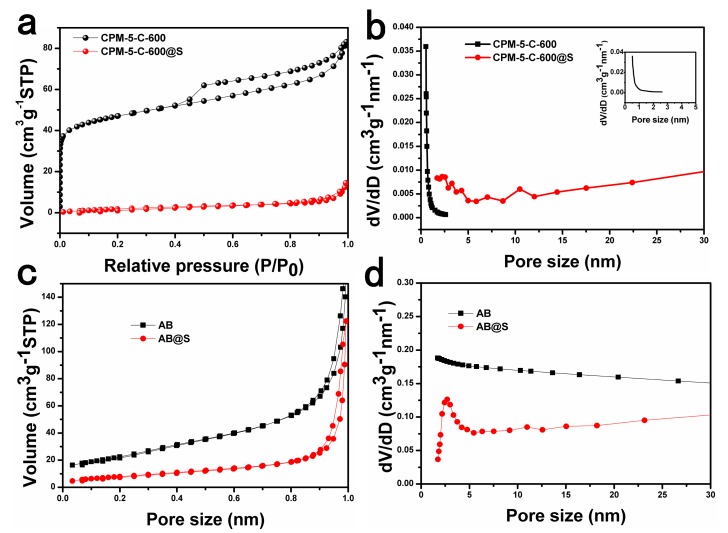
(**a**) Nitrogen adsorption-desorption isotherms and (**b**) pore size distribution curves of CPM-5-C-600 and CPM-5-C-600@S composites. (**c**) Nitrogen adsorption-desorption isotherms, and (**d**) pore size distribution curves of AB and AB@S composites.

The pore structure characteristics and pore size distribution of CPM-5-C-600, CPM-5-C-600@S, AB, and AB@S samples were listed in the figure through Brunauer-Emmett-Teller (BET) test. As shown in Figure 4a, the adsorption desorption curve of CPM-5-C-600 material shows obvious mesoporous and microporous composite structure. Through pore size analysis, we could see that there is a type of micropores (less than 1 nm) and a small amount of mesoporous (2.5 nm) on the basis of Density Functional Theory (DFT) method. Through the pore distribution structure of CPM-5-C-600, we speculate that when CPM-5-C-600@S was used as the cathode material of Li-S batteries, its microporous structure could limit the small polysulfide (S_2–4_) and prevent the transformation of small polysulfide into the large polysulfide (S_6–8_), which could improve the cycle stability of Li-S battery effectively [29]. In addition, the mesoporous structure can provide a path for the transportation of Li^+^, promote the diffusion rate of Li^+^, facilitate the infiltration of electrolyte, and elevate the conductivity and rate performance of the battery [30]. As the presence of indium oxide inhibited the adsorption of some pores on the gas, the nitrogen adsorption capacity of CPM-5-C-600 was only 80 cm^3^∙ g^−1^, which was lower than the previously reported MOF derived carbon and AB materials. And the adsorption capacity changed obviously when sulfur was poured into the material by melting diffusion. The decrease of adsorption indicates that sulfur has been successfully immersed into the pores of CPM-5-C-600, and the appearance of micro- and mesopores less than 5 nm indicates the impregnation of sulfur. The mesopores in the range of 6–20 nm could well adapt to the volume change of Li-S batteries during cycles.

**Figure 5:**
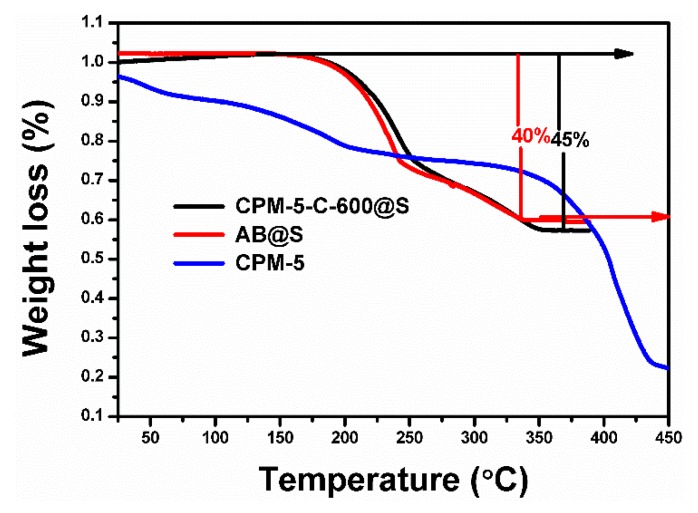
TGA curves of the CPM-5, CPM-5-C-600@S and AB@S composites.

The thermogravimetric test of CPM-5, CPM-5-C-600@S and AB@S materials were shown in the Figure 5 below. The curves of CPM-5 shows that the removal of solvent molecules occured in the temperature range of 40–230 °C and there was no further weight loss up to 300 °C. This exhibited that the CPM-5 structure had good thermal stability [21]. The carbonized CPM-5 tended to be stable above 350 °C, which also reflected its structural and thermal stability. It can be seen from the figure that the sulfur content of the two samples was 40% and 45% respectively. Both samples lost weight between 150 and 350 °C. However, by comparing the heat loss rate of two samples, we could find that the loss rate of sulfur in CPM-5-C-600@S sample was less than AB@S. This is because the bound function of CPM-5-C-600 sample to sulfur was stronger than that of AB sample, and the binding ability of sulfur to CPM-5-C-600 sample was better than that of AB sample [31]. It also provides the basis and guarantee for CPM-5-C-600 @S to be used as the cathode material of Li-S batteries, and was conducive to improve the cycle stability of the Li-S battery.

### 3.2. Electrochemical Performance

**Figure 6:**
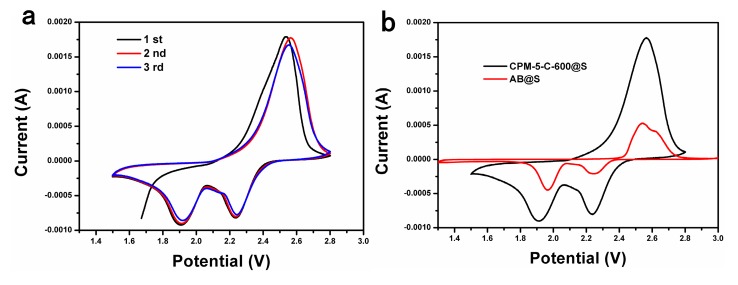
Cyclic voltammetry (CV) curves of (**a**) CPM-5-C@S electrodes at a scanning rate of 0.1 mV s^−1^ range from 1.3 to 3.0 V with 5 cycles; (**b**) CV curves of CPM-5-C@S and AB@S electrodes at the second cycle.

Within the potential range of 1.3 to 3.0 V and at a scanning rate of 0.1 mV∙s^−1^, initial three cycles CV behaviors of CPM-5-C@S electrode are shown in Figure 6a. During negative scanning, two reduction peaks centered at 1.9 and 2.25 V were observed, corresponding to the transformation of sulfur to soluble lithium polysulfides (Li_2_S_x_, 4 ≤ x ≤ 8) and soluble polysulfide to the Li_2_S_2_/Li_2_S, respectively. By contrast, the oxidation peak at about 2.5 V exhibited the oxidation reaction of Li_2_S and Li_2_S_2_ to final oxidation products of S. With the cycle processing, the intensity of the reduction peak was decreasing gradually, but the degree was low which shows the battery had strong reversibility. The curve was smooth when the sweep speed was 0.1 mV∙s^−1^, which indicates that the lithiated of the battery was weak. As shown in Figure 6b, from the growth of redox current of AB@S to CPM-5-C-600@S electrode, because the structure of CPM-5 after 600 carbonization has more electrochemical active sites than AB [32].

**Figure 7:**
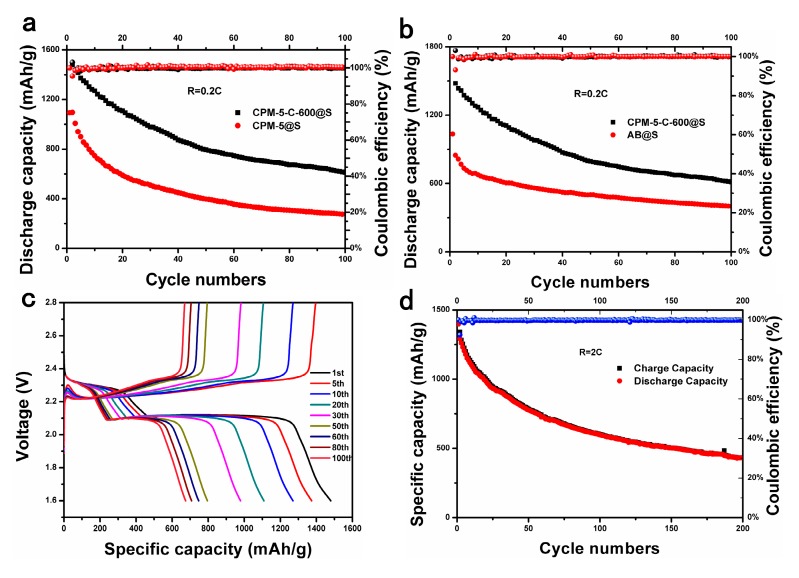
(**a**) Cycling performance of CPM-5-C-600@S and CPM-5@S electrodes at 0.2 C. (**b**) Cycling performance of CPM-5-C-600@S and AB@S electrodes at 0.2 C; (**c**) Discharge/charge curves of CPM-5-C-600@S electrodes at 0.2 C; (**d**) Cycling performance of CPM-5-C-600@S electrodes at 2 C.

In order to prove that indium oxide coated on CPM-5-C-600 contributes to inhibit the decay of polysulfide and improve the cycle stability of the battery, we used CPM-5@S and CPM-5-C-600@S as cathode of Li-S battery for 100 cycles at 0.2 C. When CPM-5@S was used as positive materials, the initial discharge specific capacity reached 1100 mAh∙g^−1^ and the capacity was maintained at 300 mAh∙g^−1^ after 100 cycles. Although the microporous of the CPM-5 could dominate the attenuation of polysulfide (S_2–4_), the capacity retention was too low due to the limitation of pore size. When CPM-5-C-600@S was used as cathode material, the first discharge specific capacity reached 1500 mAh∙g^−1^ and the ultimate capacity could be maintained at 650 mAh∙g^−1^, which shows that the presence of indium oxide did play an active role in arresting the “shuttle effect”. It slows down the dissolution by coating the spilled polysulfides, so as to increase the capacity retention rate and the cycle stability of the battery. To further evaluate the electrochemical performance of composite cathode, we compared the cycle performance of CPM-5-C-600@S and AB@S at a current density of 0.2 C as shown in Figure 7b. The high initial specific capacity of the CPM-5-C-600@S cathode assembled in the battery was much higher than the 1100 mAh·g^−1^ of the AB@S cathode. After 100 cycles, compared with 400 mAh∙g^−1^ of AB@S capacity, the high specific capacity of CPM-5-C-600@S is 650 mAh∙g^−1^, indicating good cycle stability. In addition, the constant current discharge-charge voltage profiles of the cells at 0.2 C are shown in Figure 7c. Two distinct discharge plateaus were observed for all discharge curves due to the conversion of element S_8_ to polysulfide and further reduction to Li_2_S/Li_2_S_2_ as discussed in CV measurements [33]. It can be seen from the figure that the capacity decayed quickly during the first cycle and the 50th cycle. The capacity of this part may be provided by the sulfur on the surface. In the later cycles, we could find that the capacity tended to be stable. This is the sulfur inside the channel that provides the capacity [34]. After 100 charge discharge cycles, the discharge specific capacity can still be maintained at 650 mAh∙g^−1^. The space between the curve is smaller and smaller with the cycle going on, which shows that the polarization phenomenon of the battery is weak, and the CPM-5-C-600@S has good cycling stability. The durability of the CPM-5-C-600@S electrode was further examined by charging and discharging at the current density of 2C for 200 cycles. As shown in Figure 7d, its initial discharge capacity was up to 1450 mAh∙g^−1^. After 200 cycles, it still maintained a high capacity of 500 mAh∙g^−1^, and the corresponding coulombic efficiency was about 100%. This also shows that the “Core-shell” composite formed by indium oxide and CPM-5-C-600@S buffer the volume expansion, which increases the structure stability of the positive material and elevates the constancy of the battery in the process of circulation under high current [35].

**Figure 8:**
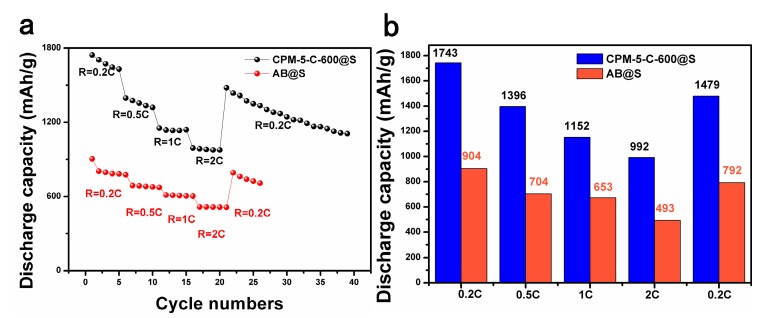
(**a**,**b**) C-rate performance for CPM-5-600@S and AB@S cathodes at various C-rate from 0.1 C to 2 C.

In order to prove that the conductivity of carbonized CPM-5 was significantly improved, we studied the rate performance of the battery. Figure 8a,b shows the rate performances of the CPM-5-C-600@S and AB@S cathodes. As the C-rate varied from 0.2 to 2 C, the discharge capacity of CPM-5-C-600@S and AB@S decreased gradually because of polarization. However, the CPM-5-C-600@S electrode was significantly higher than the AB@S electrode with the transformation of the rate. The CPM-5-C-600@S electrode could still maintain 1479 mAh·g^−1^ when the rate from 2 to 0.2 C, which was much higher than 792 mAh·g^−1^ of AB@S electrode. CPM-5-C-600@S electrode shows good conductivity and excellent cycle stability. We compared the electrochemical performance of the CPM-5-C-600@S cathode in this work with other related reported composite material derived from MOF. The results were summarized in Table 1.

**Figure 9:**
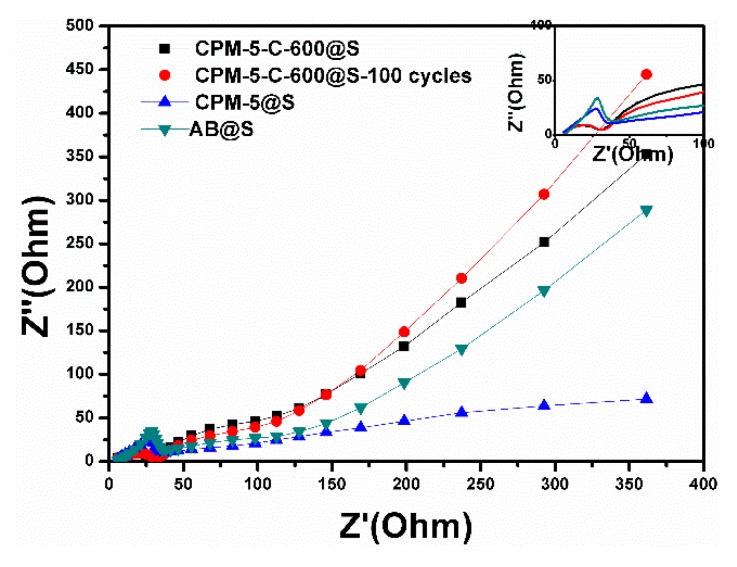
The EIS of AB@S, CPM-5@S, CPM-5-C600@S electrodes in Li-S batteries before cycles and the CPM-5-C-600@S-100 cycles electrodes.

The EIS curves of AB@S, CPM-5@S, CPM-5-C-600@S, and CPM-5-C-600@S-100 cycles cathodes are showed in Figure 9. The semicircle could reflect the resistance of the electrochemical reaction at the boundary of the electrode-electrolyte, which is called charge-transfer resistance [36]. We could see that the transfer resistance of CPM-5-C-600@S electrode material was less than that of AB@S and CPM-5@S electrode, which attributes to the special structure of CPM-5-C-600. On the other hand, the Li^+^ diffusion coefficient (slope of oblique line) of CPM-5-C@S electrode was larger than that of AB@S and CPM-5@S electrode, because the mesopores of CPM-5-C-600 structure increased the diffusion rate of Li^+^ and improves the conductivity of the battery [37,38]. After 100 charge discharge cycles at 0.2 C, the cell impedance remained stable with CPM-5-C-600@S cathode. During the discharge process, the solid sulfur was reduced to polysulfide and deposited on the carrier material. This will promote the transfer impedance between polysulfides and the carrier material, but the structure of carbonized CPM-5can effectively avoided this defect. The stability of the structure, micro- mesoporous combination and indium oxide particles made the composite material cathode reduce the deposition of lithium sulfide and the obstruction of Li^+^ diffusion process, so as to improve the conductivity of the battery [39,40].

## 4. Conclusions

In summary, CPM-5-C-600 was successfully obtained by hydrothermal and carbonization methods. Its unique micro-mesoporous composite structure and indium oxide solid particles obtained by carbonization were conducive to inhibiting the decay of polysulfide and improving the rate performance and cycle stability of the battery. When CPM-5-C-600@S was used as cathode a material of Li-S battery, the capacity was of 650 mAh·g^−1^ at 0.2 C of 100 cycles and 500 mAh·g^−1^ at 2 C of 200 cycles respectively. The coulombic efficiency was close to 100%.

## Figures and Tables

**Table 1:** Electrochemical performances of CPM-5-C-600@S electrode in this work and other similar composition reported in other references as cathode in Li-S battery.

Samples	Initial Capacity	Cycling Stability	Rate Capacity	Reference
CPM-5-600-C@S	1500 mAh g^−1^	794 mAh g^−1^ at 0.2 C after 50 cycles 650 mAh g^−1^ after 100 cycles	992 mAh g^−1^ at 2 C	this work
CoS/KB	1500 mAh g^−1^	600 mAh g^−1^ at 0.5 C after 200 cycles	700 mAh g-1 at 2 C	[16]
MOF-74/CNT	1065 mAh g^−1^	610 mAh g^−1^ at 0.5 C after 100 cycles	667 mAh g^−1^ at 2 C	[38]
Fe_3_O_4_/C/S	1050 mAh g^−1^	750 mAh g^−1^ at 0.2 C after 50 cycles	640 mAh g^−1^ at 1 C	[31]
HPCN-S(MOF-5)	1177 mAh g^−1^	730 mAh g ^−1^ at 0.5 C after 50 cycles	/	[30]
C from ZnFumarate	1500 mAh g^−1^	650 mAh g^−1^ at 400 mA g^−1^ after 40 cycles	/	[22]
MIL-101@rGO/S	980 mAh g^−1^	650 mAh g^−1^ at 335 mA g^−1^ after 50 cycles	980 mAh g^−1^ at 0.2 C	[39]
S/Gd2(Gd_2_O_3_)-CA	1210 mAh g^−1^	555 mAh g^−1^ at 0.1 C after 50 cycles	420 mAh g^−1^ at 1 C	[24]
S/FLHPC	1206 mAh g^−1^	856 mAh g^−1^ at 0.2 C after 100 cycles	763 mAh g^−1^ at 2 C	[27]
